# Editorial: Novel intervention models transcending borders for stress management - volume III

**DOI:** 10.3389/fpsyg.2026.1845466

**Published:** 2026-04-21

**Authors:** Adelinda Araujo Candeias, Mariola Bidzan, Konrad Reschke, Marcus Stueck, Edgar Galindo

**Affiliations:** 1 University of Evora, Évora, Portugal; 2 Uniwersytet Gdanski, Gdańsk, Poland; 3 Universitat Leipzig, Leipzig, Germany; 4 International Biocentric Research Academy (IBRA), Leipzig, Germany; 5 Comprehensive Health Research Centre Polo de Evora, Evora, Portugal

**Keywords:** biopsychosocial approach, ecological intervention, mental health, preventive intervention, stress management, wellbeing

This book represents the third volume of the Research Topic *The Interplay of Stress, Health, and Well-being: Unraveling the Psychological and Physiological Processes*. Building on the foundations laid by the previous volumes, this edition sought to further broaden the field by incorporating new empirical insights, fresh conceptual perspectives, and innovative forms of intervention. The strong response from the scientific community once again reflects the growing relevance of stress research across disciplines, settings, and cultural contexts. In continuity with the scientific and editorial direction established in the previous volumes, this third Research Topic further consolidates the idea that stress research can no longer be confined to a single disciplinary domain. Rather, it has become a genuinely interdisciplinary and international field of inquiry, requiring the articulation of psychological, physiological, social, cultural, educational, and contextual levels of analysis (Candeias et al., 2024, 2025).

The collection of works presented here reinforces an idea already expressed multiple times in previous issues: stress is a phenomenon that clearly affects mental health and wellbeing and is associated with anxiety, depression, fatigue, and numerous health problems, including pain, cardiovascular conditions, and musculoskeletal disorders (Candeias et al., 2024; Reschke and Mätzchen, 2020). At the same time, the studies gathered in this issue confirm that stress cannot be fully understood solely through physiological and psychological processes. Rather, it is also shaped by personal factors, such as optimism, coping resources, and self-regulation capacities, as well as by social, relational, developmental, and cultural dimensions, all of which influence both vulnerability and resilience (Candeias et al., 2025; Galindo et al., 2022). Contemporary health hazards are increasingly complex, and stress itself has become a multifaceted construct, shaped by rapid temporal change, macrosocial transitions, digital acceleration, uncertainty, and overlapping crises that affect individuals and communities in cumulative ways (Candeias et al., 2021). In this context, individual and collective capacities for stress resistance and stress management require both established and innovative intervention approaches capable of responding to changing ecological, occupational, educational, and psychosocial demands. The concept of adaptive intelligence (Sternberg, 2021) is therefore highly relevant and deserves further consideration within the field of stress research, particularly because it emphasizes flexibility, context-sensitive decision-making, and the capacity to respond constructively to instability and complexity.

A central contribution of this volume lies in the way it brings together studies that, although diverse in topic and methodology, converge on a common message: stress is not merely an individual reaction, but a dynamic process emerging from the interaction between persons, contexts, life transitions, social structures, and cultural frameworks. In this regard, many of the contributions examine the role of already recognized stressors while advancing our understanding of the protective and moderating influence of social and community support (Lu et al.; Resch and Bellhäuser; Nsia et al.; Uditha and Bulathwatta), the impact of gender and developmental variables (Candeias A. A. et al.), and the importance of cultural context (Wang et al.; Candeias A. et al.; Alhasan et al.). Together, these studies make clear that stress is neither produced nor regulated in a social vacuum. It is shaped by social relations, institutional environments, developmental demands, and culturally embedded ways of interpreting adversity, coping, and support. As previous work has already shown, psychological adjustment, quality of life, and wellbeing are significantly influenced by broader contextual conditions, including public health crises, social inequalities, and institutional responses to uncertainty (Candeias et al., 2021).

From this perspective, one of the most relevant conceptual contributions of the volume is the reinforcement of a biopsychosocial understanding of stress and health. The articles collectively suggest that health cannot be adequately understood as the mere absence of symptoms, nor can mental health be reduced to the treatment of distress after it emerges. Instead, health appears here as a dynamic, relational, and context-dependent process involving biological regulation, psychological adaptation, social connectedness, meaning-making, and the availability of protective resources in everyday life. This view has important implications for contemporary health sciences, as it supports more integrative and ecologically grounded approaches to prevention and intervention. It encourages a move beyond fragmented models and toward forms of care and health promotion that are more sensitive to lived contexts, more responsive to diversity, and more capable of integrating individual, interpersonal, institutional, and community dimensions. It is also consistent with broader perspectives emphasizing the embodied and integrated nature of intelligence, consciousness, emotion, and adaptive regulation in human functioning (Damasio, 2026).

One of the greatest strengths of this new volume lies precisely in the breadth of its empirical and cultural scope. The articles cover diverse geographical and sociocultural contexts, including Europe, the Middle East, North Africa, East Asia, South Asia, and North America, thus reinforcing the importance of viewing stress not only as a universal human experience, but also as one that is expressed, interpreted, and managed through culturally embedded frameworks. This international breadth contributes to a richer and more nuanced understanding of stress and emotional health, while also supporting the development of intervention models that are both evidence-based and context-sensitive. In this sense, transcending borders means not only comparing populations across countries, but also recognizing the plurality of psychosocial realities in which stress emerges, acquires meaning, and becomes a target for prevention or intervention. It also means acknowledging that similar stressors may be experienced, interpreted, and managed differently according to cultural expectations, social support systems, institutional resources, and developmental conditions.

Another important strength of this collection is its applied orientation. Most of the articles do not merely describe stress-related problems; they are also explicitly concerned with practical implications, whether through the development of intervention programs, the improvement of institutional policies, or the design of clinical, educational, organizational, and community-based applications. This translational orientation is especially relevant in a field where research findings are increasingly expected to inform prevention, health promotion, and intervention strategies across multiple sectors. Indeed, previous evidence has already shown that stress management programs can produce meaningful benefits in professional and care settings, contributing not only to reduced distress but also to improved adaptation and wellbeing (Galindo et al., 2020). The present volume extends this applied perspective by presenting interventions that are diverse in format, population, and setting, thereby illustrating how stress management may be operationalized through multiple modalities and tailored to the needs of distinct groups. More importantly, it shows that intervention is not restricted to the clinical treatment of distress, but may also involve preventive action, educational innovation, community support, and the strengthening of personal and collective resources.

In this context, the articles gathered here cover a diverse range of intervention approaches, including meditation, mindfulness, yoga, Reiki, and creative dance (Nakano; Mohamed et al.; Bao et al.; Joss et al.; Yan et al.; Han et al.; Bablis et al.); emotional regulation and interoceptive awareness (Rosado et al.; Crivelli et al.; Mohamed et al.); high-intensity interval training (Bao et al.); physical activity (Wang et al.); as well as psychosocial interventions (Nsia et al.), social support (Lu et al.; Resch and Bellhäuser), and the use of modern technologies such as online mindfulness (Joss et al.), computer-based approaches (Candeias A. et al.), and videoconferencing training (Boß et al.). This diversity is particularly valuable because it reflects the multifactorial nature of stress itself and the corresponding need for intervention models that are not restricted to a single therapeutic tradition or methodological approach. Instead, the studies in this volume suggest that effective responses to stress may emerge from the articulation of body-based practices, emotional and cognitive regulation, interpersonal resources, educational pathways, and technology-mediated formats. Collectively, these contributions encourage a broader view of intervention—one that recognizes multiple entry points for action and values flexibility, accessibility, and adaptation to context.

Importantly, this broader view also contributes to a more innovative understanding of mental health intervention. Innovation, in this sense, does not lie only in the introduction of new techniques or tools, but in the capacity to redesign intervention models so that they are more preventive, more ecological, more sustainable, and more interdisciplinary. A preventive model seeks to act before distress becomes chronic or disabling. An ecological model recognizes that mental health is shaped in schools, workplaces, families, communities, and digital environments, and not only in clinical settings. A sustainable model privileges interventions that can be integrated into everyday life, disseminated across contexts, adapted to available resources, and maintained over time. An interdisciplinary model accepts that meaningful progress in mental health promotion requires dialogue among psychology, health sciences, education, occupational health, public health, and technology. In this sense, the volume offers not only empirical findings but also a conceptual orientation for rethinking mental health care in contemporary societies.

Several contributions also illustrate how intervention and prevention may be strengthened when stress is approached not merely as a problem to be reduced, but also as a process that can be better understood, regulated, and transformed through learning and adaptation. Some studies emphasize the role of social and relational resources; others focus on behavioral, emotional, or embodied strategies; still others underline the value of educational and technological approaches capable of reaching broader populations and supporting mental health promotion in accessible ways. Within this broader framework, the perspective advanced in previous work (Galindo et al., 2022) is further expanded and reinforced, namely the idea that stress management should also be understood as a constructive and educable process, involving the promotion of adaptive resources, positive coping orientations, and sustainable forms of self-regulation. This view is especially valuable in the present volume, where several studies move beyond the identification of symptoms or risks and instead emphasize capacities for learning, adaptation, and resilience across different contexts and populations. In this respect, the volume contributes to a more developmental and empowering vision of stress management, one that is less centered on deficit alone and more attentive to competence-building, protective resources, and the possibility of positive transformation.

From a health perspective, this shift is especially significant. It suggests that innovation in mental health should not be measured only by technological sophistication or therapeutic novelty, but also by the extent to which interventions are capable of being integrated into lived environments, promoting agency, strengthening social bonds, and supporting healthier modes of adaptation. The biopsychosocial perspective underlying many of the contributions encourages a form of health intervention that is closer to people's realities, more attentive to complexity, and better aligned with the long-term goals of wellbeing, participation, resilience, and quality of life. Such an approach is particularly important at a time when health systems are challenged by chronic stress, mental overload, widening inequalities, and the need for scalable yet human-centered responses.

With regard to the target population, it is evident the researchers' interest in vulnerable groups such as health professionals (Alhasan et al.; Nsia et al.; Han et al.; Crivelli et al.), workers in general (Nakano; Resch and Bellhäuser; Boß et al.; Litan), clinical patients (Lu et al.; Joss et al.), vulnerable families and communities (Uditha and Bulathwatta; McCutcheon and Habiya), and older adults (Rosado et al.). Interest in university students also remains clearly present (Elsayed; Yan et al.; Bao et al.), while other contributions broaden the developmental perspective by considering adolescents and young adults and by examining how variables such as age, gender, socio-emotional competencies, and health-related behaviors shape wellbeing and vulnerability profiles (Candeias A. A. et al.). Taken together, these studies reinforce a life-span view of stress and wellbeing, showing that both risk and protection must be understood in relation to developmental stage, contextual demands, and available psychosocial resources. They also suggest that prevention and intervention should be responsive not only to clinical symptoms, but also to the specific educational, occupational, familial, and social ecologies in which stress is experienced. This life-span perspective is especially important because it reminds us that stress-related challenges do not affect all groups in the same way, nor do they call for identical forms of support, prevention, or intervention.

More broadly, the articles in this volume show that stress management requires flexible, integrated, and culturally sensitive approaches capable of connecting psychology, public health, occupational health, education, and technology (Galindo et al., 2020, 2022). They demonstrate that transcending borders in stress research means not only crossing geographical boundaries, but also building bridges between disciplines, methods, developmental stages, and intervention frameworks. This is one of the key messages emerging across the three volumes of this Research Topic: understanding stress in contemporary societies requires multi-level models that integrate biological processes, subjective experience, interpersonal relations, institutional structures, and cultural meaning systems (Candeias et al., 2024, 2025). In this sense, Volume III offers a meaningful contribution to the advancement of international and interdisciplinary knowledge on stress, health, and wellbeing, while also pointing toward practical and adaptable pathways for intervention in contemporary societies. It highlights that innovative intervention models are most promising when they are scientifically grounded, contextually sensitive, and open to dialogue across borders—geographical, disciplinary, developmental, and methodological. As such, this volume not only consolidates previous advances in the field, but also opens new avenues for future research and action aimed at promoting healthier, more resilient, and more adaptive individuals and communities.
